# Shark Spotters: Successfully reducing spatial overlap between white sharks (*Carcharodon carcharias*) and recreational water users in False Bay, South Africa

**DOI:** 10.1371/journal.pone.0185335

**Published:** 2017-09-25

**Authors:** Tamlyn Engelbrecht, Alison Kock, Sarah Waries, M. Justin O’Riain

**Affiliations:** 1 Institute for Communities and Wildlife in Africa, Department of Biological Sciences, University of Cape Town, Cape Town, South Africa; 2 Shark Spotters, Cape Town, South Africa; 3 South African National Parks, Cape Research Centre, Cape Town, South Africa; 4 South African Institute for Aquatic Biodiversity (SAIAB), Grahamstown, South Africa; Department of Agriculture and Water Resources, AUSTRALIA

## Abstract

White sharks (*Carcharodon carcharias*) are apex predators that play an important role in the structure and stability of marine ecosystems. Despite their ecological importance and protected status, white sharks are still subject to lethal control to reduce the risk of shark bites for recreational water users. The Shark Spotters program, pioneered in Cape Town, South Africa, provides a non-lethal alternative for reducing the risk of human-shark conflict. In this study we assessed the efficacy of the Shark Spotters program in reducing overlap between water users and white sharks at two popular beaches in False Bay, South Africa. We investigated seasonal and diel patterns in water use and shark presence at each beach, and thereafter quantified the impact of different shark warnings from shark spotters on water user abundance. We also assessed the impact of a fatal shark incident on patterns of water use. Our results revealed striking diel and seasonal overlap between white sharks and water users at both beaches. Despite this, there was a low rate of shark-human incidents (0.5/annum) which we attribute partly to the success of the Shark Spotters program. Shark spotters use visual (coloured flags) and auditory (siren) cues to inform water users of risk associated with white shark presence in the surf zone. Our results showed that the highest risk category (denoted by a white flag and accompanying siren) caused a significant reduction in water user abundance; however the secondary risk category (denoted by a red flag with no siren) had no significant effect on water users. A fatal shark incident was shown to negatively impact the number of water users present for at least three months following the incident. Our results indicate that the Shark Spotters program effectively reduces spatial overlap between white sharks and water users when the risk of conflict is highest.

## Introduction

Human-wildlife conflict presents one of the greatest challenges to the effective conservation of wildlife species [1–4]. Typically, the response to this form of conflict is the lethal control of problematic species, a practice which poses a risk to threatened species, as well as the stability and health of natural systems [1,5]. Apex predators are most at risk to lethal control as these animals pose an indirect threat to human livelihoods through predation on livestock or game animals and a direct threat to humans through rare, but severe or fatal attacks [5–7]. Furthermore, the life history characteristics typical of apex predators such as slow growth, late age of sexual maturity, low reproductive capacity and low overall abundance has made them particularly vulnerable to extensive lethal control [8–10]. Apex predators play an important regulatory role in trophic networks, and numerous studies have documented the severe and unpredictable cascading effects of removing these top order predators from the ecosystems they inhabit [11–16].

White sharks are apex predators that are subject to lethal control as a consequence of infrequent, but severe or fatal attacks on humans. In the period from 1839–2010 there have been a total of 346 unprovoked white shark attacks (102 fatalities) recorded worldwide (an average of less than two attacks per year) [17]. “Unprovoked” attacks refer to those in which humans are bitten due to overlap with sharks in their natural environment, with no prior provocation of the shark, whereas “provoked” attacks are those in which humans attempt to touch or handle sharks (such as during scuba diving or hook removal from fishing) and are subsequently bitten [17]. The “low-probability, high consequence” nature of unprovoked shark attacks skew people’s risk perception, and these incidents have been shown to lead to overreaction by the public and policy makers [18–20]. A number of areas with sustained high levels of shark attacks have implemented lethal control strategies to lower the risk of human-shark conflict and restore public confidence in beach safety. These measures are varied, and range from short-term shark hunts and culls, to long-term control methods, including the use of permanently or semi-permanently deployed fishing gear such as large-mesh gill nets and/or baited “drum lines” [17]. There is substantial debate as to the efficacy of lethal control measures, and several studies have shown that short-term, concentrated culls are largely ineffective at reducing the risk of shark bites from large, wide ranging species such as white sharks [21,22]. However, long-term shark control programs, such as those currently implemented at a number of popular beaches in Queensland and New South Wales in Australia, and in KwaZulu Natal, South Africa, have been shown to successfully reduce the number of shark incidents in these areas [23–25]. Unfortunately, these methods are costly in terms of their environmental impacts. Not only do shark nets and drum lines reduce the numbers of a protected apex predator, nets in particular are also highly unselective, resulting in the by-catch of numerous species including cetaceans, rays, turtles and a number of harmless shark species [25–27]. Alternative, non-lethal strategies that can be implemented on a long-term basis are therefore needed to mitigate conflict between water users and sharks.

In False Bay, South Africa, a novel community initiative, Shark Spotters, has been implemented as an alternative to lethal methods [20]. The proximity of mountains to a number of popular beaches provides vantage points from which trained shark spotters can alert the public to the presence of white sharks in, or close to, the surf zone using a combination of auditory (a siren) and visual (flags) warnings [20]. Shark Spotters aims to balance the needs of recreational water users in Cape Town with the conservation of white sharks by actively reducing overlap between people and sharks in the inshore zone and hence mitigating potential conflict [20]. However, the success of the program relies strongly upon the cooperation and compliance of water users with warnings issued by spotters in the event of a shark sighting.

In this study we assess the extent of spatial overlap between white sharks and recreational water users in the inshore zone of two popular beaches in False Bay. We also assess the impact of visual and auditory warnings issued by the spotters on the abundance of different water users (surfers, paddlers and bathers, respectively). Finally, we investigate the change in water user abundance in the months following the occurrence of a fatal attack, in order to determine the impact of such an event on patterns of water use.

## Methods

### Study sites

**Fig 1:**
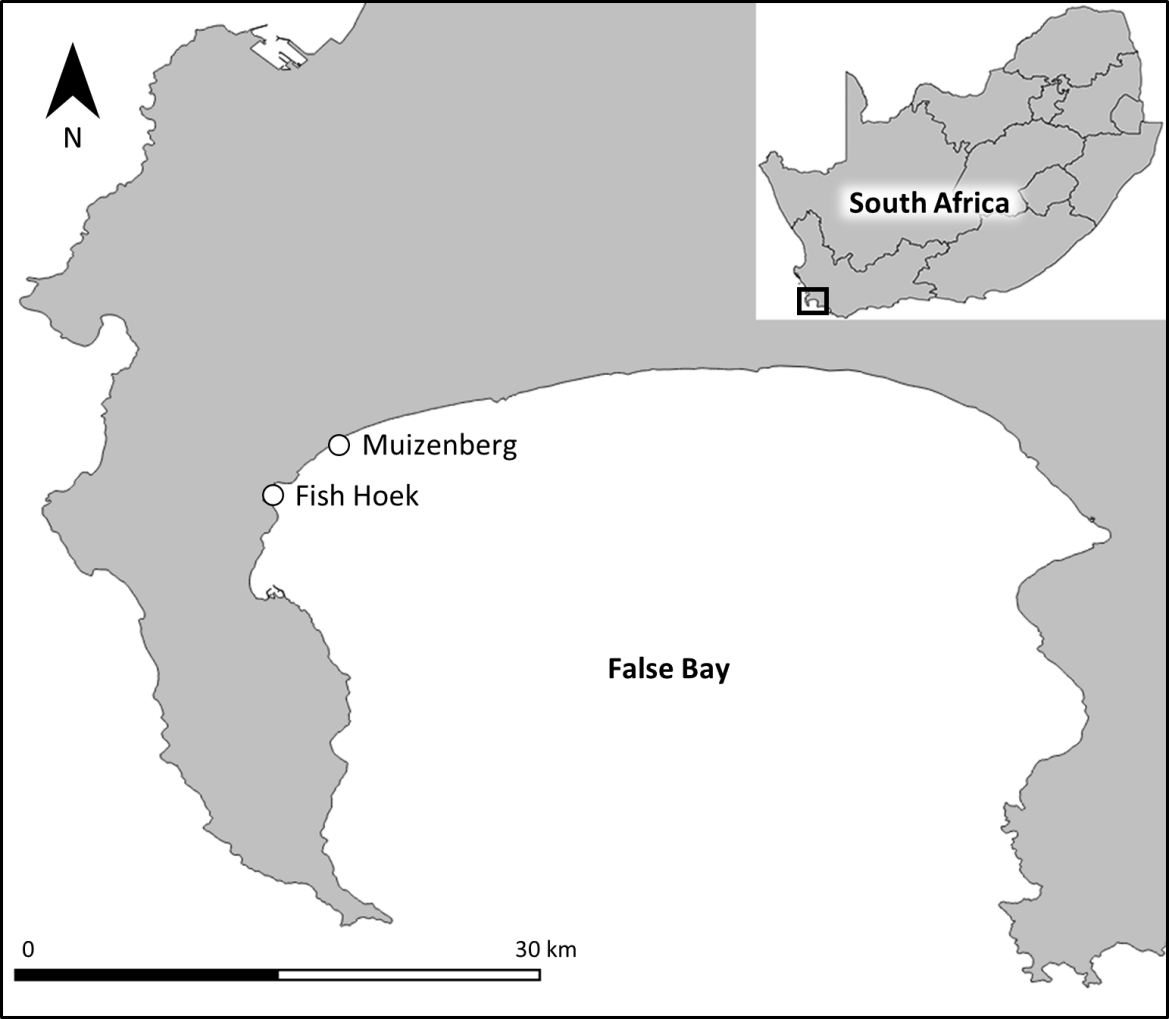
Locations of Muizenberg and Fish Hoek beaches in False Bay, South Africa.

The Shark Spotters program operates at eight beaches around False Bay and the Cape Peninsula, located in the Western Cape province of South Africa. This is in accordance with a memorandum of understanding with the City of Cape Town municipality, which became a formal partner of the program in 2006. Of these eight beaches, four have spotters present 365 days a year (permanent beaches), while the other four only have spotters in place during spring/summer (seasonal beaches). This study made use of data collected by shark spotters from two of the most popular permanent beaches, namely Fish Hoek and Muizenberg (Fig 1). Although these beaches are in close proximity, they differ in bathymetry and hence wave action, which strongly influences the dominant water user groups that frequent each beach. Muizenberg is a gently sloping beach characterised by an extended surf zone (>300 m from the beach), and is a popular beach for surfers. Fish Hoek is more steeply sloped with a narrow surf zone (<50 m from the beach), and is predominantly frequented by bathers and kayakers.

Spotters make use of vantage points on mountains adjacent to each beach (approximately 120 m above sea level at Fish Hoek and 100 m at Muizenberg) to detect sharks. Spotters then communicate their sightings to colleagues on the beach who operate a flag and siren system to inform water users of the potential risk from white sharks. While the majority of sharks sighted are white sharks, other species are occasionally sighted in the inshore regions of False Bay in the summer months, including bronze whalers, hammerheads and thresher sharks. These sharks are usually distinguishable from white sharks due to their smaller size, their distinct swimming behaviour or body shapes, as well as their tendency to be spotted in small groups rather than individually [20]. Independent verification of species identification opportunistically using boat-based observers, as well as camera and video footage of sharks sighted on 23 separate occasions, confirmed that the species had been correctly identified by shark spotters [20]. However, adverse spotting conditions and the distance of the shark from the spotter’s position can make species identification challenging. In these instances the Shark Spotters’ protocol and warning procedure state that any large shark sighted in close proximity to water users is sufficient cause to warn the water users.

**Fig 2:**
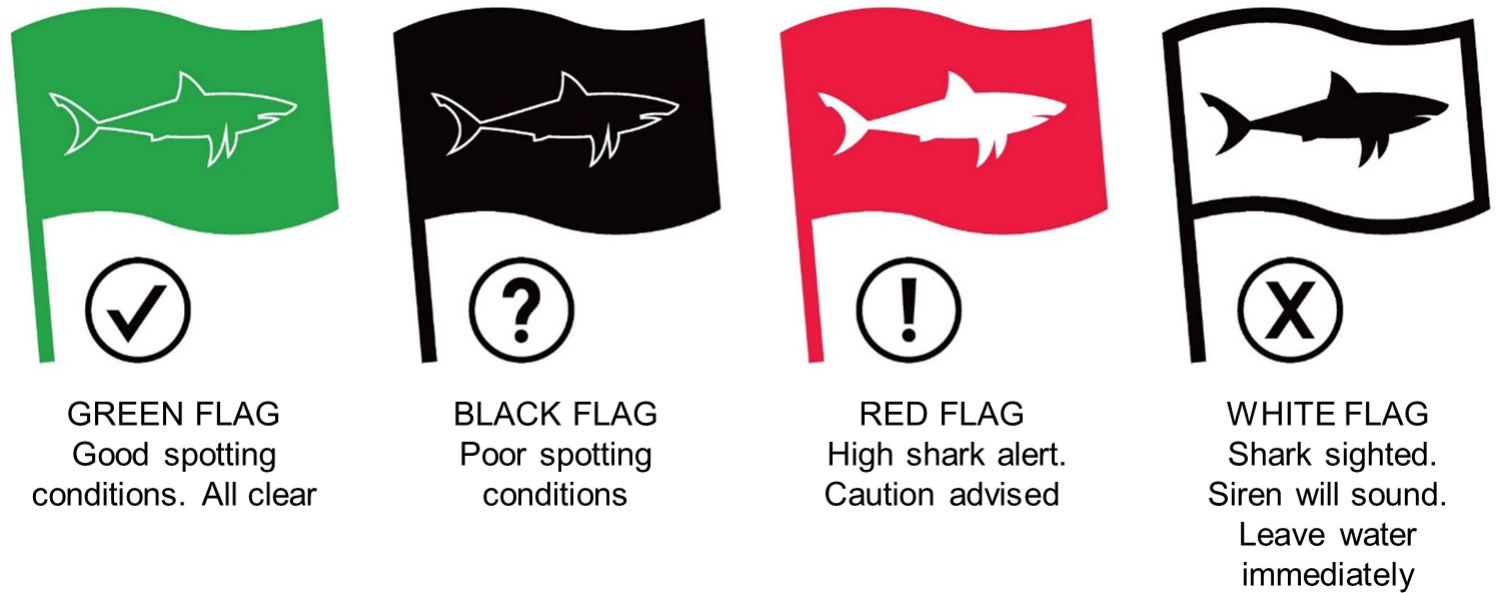
Flag system used by Shark Spotters to advise water users of spotting conditions, shark sightings and risk level when entering the water.

The program uses a four-flag system and siren to communicate spotting conditions, shark sightings and potential risk to the public (Fig 2). Only the white flag is accompanied by a loud siren, clearly audible to water users. When a shark is sighted in close proximity to water users, the siren will sound repeatedly and shark spotters will actively encourage all water users to clear the water. Thereafter, the beach remains temporarily closed and spotters will actively discourage people from re-entering the water until the shark has moved out of the surf zone. The red flag (“High shark alert”) is raised under a number of different circumstances, including: when a shark is sighted well beyond the surf zone and poses no direct threat to water users; for the hour directly following a beach closure to warn the public that a shark was recently sighted in the surf zone; when activities in close proximity to public beaches may be conducive to increased shark presence in the inshore zone (e.g. trek-netting, the presence of game fish or a whale stranding).The red flag is raised without an accompanying siren or active beach clearing by spotters, and does not result in a temporary beach closure. This flag serves to warn water users of increased risk of shark presence in the surf zone; however the decision to enter/remain in the water remains at the discretion of the public.

### Data collection by shark spotters

Shark spotting duty is divided into two shifts, a morning shift from 08h00–13h00 (07h00 in summer for Fish Hoek), and an afternoon shift from 13h00–18h00 (19h00 in summer for Fish Hoek). The number of different water users (surfers, paddlers and bathers) and the corresponding flag colour are recorded on the hour, for every hour throughout each shift. Shark spotters use one of two methods to determine the number of water users present during each hourly count: either via a direct census (when water user numbers are low), or a simplified version of the “Jacob’s method” of crowd size estimation (for larger numbers of water users) [28]. The latter method involves counting a small section of water users and extrapolating this value to get an estimate of the total number of water users present.

The total number of shark sightings per shift is recorded, and for each sighting the spotters record the time the shark was first seen, last seen, whether the shark entered the surf zone (resulting in the white flag being raised with an accompanying siren), or if the shark was sighted well beyond the backline and did not pose an immediate threat to water users (resulting in the red flag being raised with no accompanying siren).

### Data analyses

All white shark sightings from 2006–2014, and all hourly water use data from 2007–2014 (due to incomplete water use records prior to 2007) were included in analyses (Statistica v11, 2012).

#### Influence of poor visibility on shark sightings

Preliminary data exploration indicated a high incidence (>90% of spotting hours at both beaches over the study period) of “poor visibility” conditions for spotters attempting to detect sharks, i.e. the black flag was flying. Despite this, the majority of all shark sightings (>85% across both beaches) were recorded in “poor visibility” conditions. We thus used all sightings data irrespective of environmental conditions that influence visibility, acknowledging that the number of sightings reported will therefore be a minimum estimate. Importantly, spotting effort was constant across seasons and years.

#### Temporal patterns in shark sightings and recreational water use

In order to investigate seasonal patterns in white shark sightings for each beach, the total number of shark sightings per month was averaged over the period 2006–2014. This was only done for sightings in which the species was identified by the spotter as a white shark. However, we acknowledge that errors in species identification may still have occurred in adverse spotting conditions, and hence there is the possibility of small error margins on white shark counts along the inshore.

The number of water users present on an hourly basis was then averaged for each month over the period 2007–2014 in order to investigate the extent of overlap between white sharks and humans in the inshore zone on a monthly basis. Due to the difficulty of reliably estimating water user numbers in a dynamic and extensive system such as the inshore coastal region, we acknowledge that absolute estimates of water user abundance will differ among spotters. However, the fact that spotters are designated to a particular beach permanently over multiple seasons means that the margin of error across days and months can be assumed to be fairly constant. As neither data set was normally distributed, non-parametric Kruskal Wallis ANOVA’s (95% confidence level) were used, followed by post hoc comparisons of mean ranks of all pairs of groups (post hoc Dunn’s test) in order to determine if there were significant differences in the number of shark sightings and water users according to month.

We then assessed spatial overlap between white sharks and water users on a finer scale, by investigating diel trends in the number of shark sightings and water users. The number of shark sightings that occurred at each hour of the day (08h00–18h00) was averaged annually over the period from 2006–2014. A cube root transformation was used to normalise the data and following this a one-way ANOVA (followed by post-hoc Tukey’s test) was used to determine if time of day had a significant impact on average number of shark sightings. The impact of time of day on the average hourly number of water users was analysed at each beach for each water user group using a non-parametric Kruskal Wallis ANOVA followed by post hoc comparisons of mean ranks of all pairs of groups (post hoc Dunn’s test). This allowed a comparison of trends in white shark presence and recreational water use in the inshore zone on an hourly basis, with the aim of determining which times of day overlap between people and sharks was highest.

#### Impact of shark warnings on different water user categories

The number of water users present in each water user category is recorded on the hour, for every hour the spotters are on duty. We analysed the change in water user abundance in each category over two consecutive hourly counts for cases in which there was no shark present during the first count, but there was an ongoing sighting at the time of the second count. This analysis was done separately for sightings where the white flag and siren were used to warn water users of shark presence (high risk sightings) and sightings where only the red flag was used as a warning signal with no accompanying auditory cue (lower risk sightings), in order to compare the impact of different warning procedures on water user abundance.

This limited the number of sighting records that could be analysed (as the shark had to still be present at the time of the second count), and hence comparisons for each water user category were averaged across both beaches. The narrow time frame of 60 minutes between consecutive counts allowed the exclusion of numerous confounding variables (e.g. weather, water temperature, different spotters) that could influence water user counts. As the data were not normally distributed, comparisons between consecutive counts for each water user group were conducted with Mann-Whitney U tests.

#### Impact of consecutive flag changes on water user abundance

When a shark is present in the surf zone the white flag will be kept in place and the beach closed until the shark moves out of visual range of the spotter. Once the spotter is confident the shark has left the surf zone the beach will be re-opened with the red flag in place to advise extreme caution due to the unpredictable nature of sharks and the inability of spotters to guarantee that they can detect the shark at all times. What is unclear is how water users respond to these successive changes in flags. In order to assess this, we analysed the change in mean water user abundance over three consecutive hourly counts, where the black or green flag was flying during the first count, the white flag during the second count, and the red flag during the third. This was done for each water user category, averaged across both beaches. Comparisons were done separately for each water user category using Kruskal Wallis ANOVAs followed by post hoc comparisons of mean ranks of all pairs of groups (post hoc Dunn’s test).

#### Impact of a fatal attack on water user abundance

In order to investigate the impact of a fatal attack on water use patterns we compared the average hourly number of water users present across both beaches and all water user categories for the three month period before (11 October 2009–11 January 2010) and after (13 January 2010–13 April 2010) a fatal attack occurred at Fish Hoek beach. We compared these data to the change in the average number of water users over the same period in years during which no attack took place at either beach (2007, 2008, 2012 and 2013) to account for seasonal variation in water user abundance. The difference in average number of water users was compared using a Mann-Whitney U test.

## Results

### Seasonal patterns in shark sightings and recreational water use

#### Shark sightings

**Fig 3:**
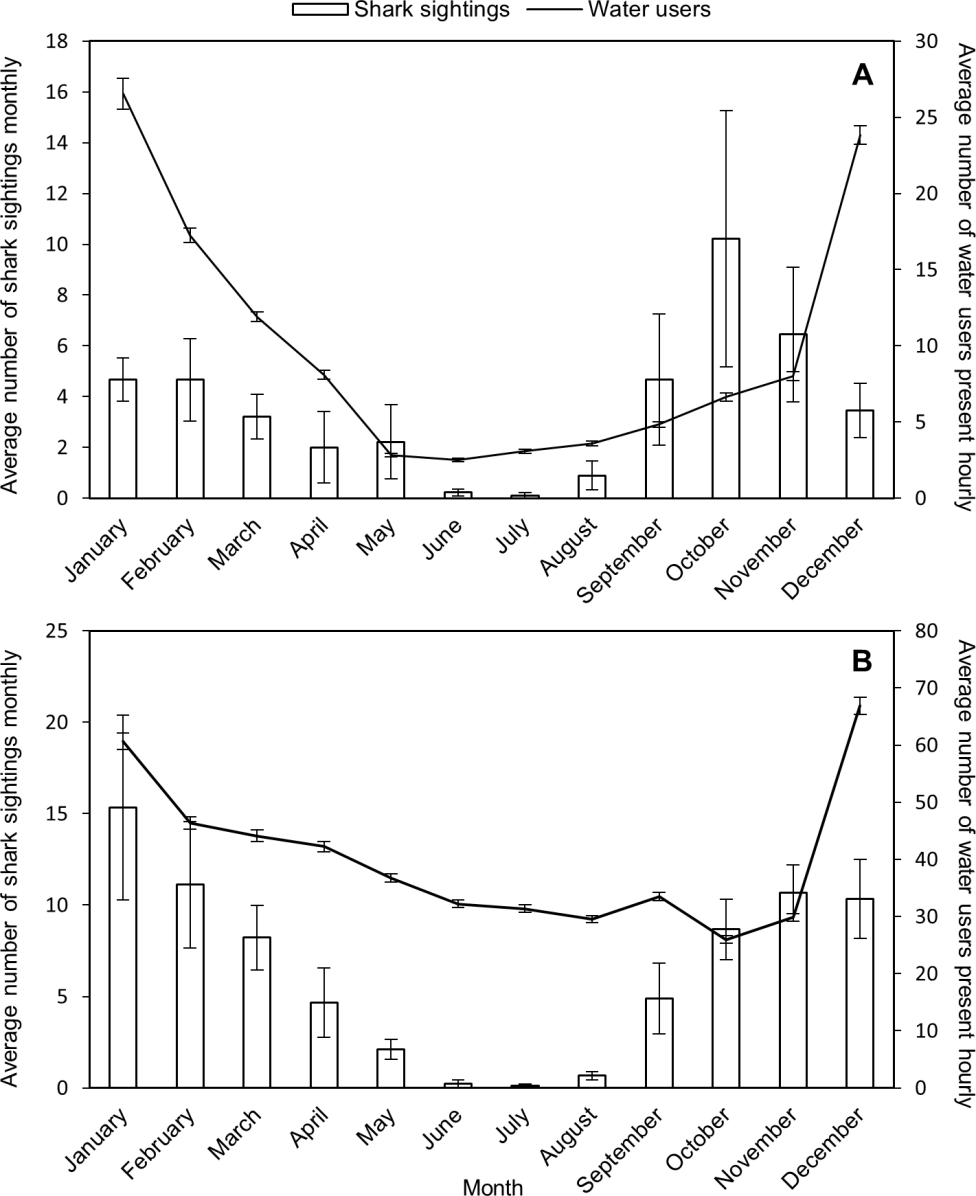
Average number (±SE) of monthly shark sightings (bars) and all water users (surfers, paddlers, bathers–solid line) present hourly across all study years at (A) Fish Hoek beach, and (B) Muizenberg beach.

At Fish Hoek, the average number of shark sightings varied significantly across months (H = 36.64, N = 108, p = 0.0001, Fig 3A) with the austral spring and summer months of October and January having significantly more sightings (post hoc Dunn’s test: p < 0.05) than the winter months of June and July. Similar to Fish Hoek, the average number of shark sightings at Muizenberg varied significantly seasonally (H = 63.84, N = 108, p < 0.001, Fig 3B), with the spring and summer months of November, December, January and February having significantly more sightings (post hoc Dunn’s test: p < 0.05) than the winter months of June, July and August.

#### Recreational water users

At Fish Hoek the average number of water users present hourly varied significantly across months (H = 5139.32, N = 32321, p < 0.001, Fig 3A), with significantly fewer water users in the autumn and winter months of May–August relative to the rest of the year (post hoc Dunn’s test: p < 0.001). Similarly, at Muizenberg the average number of water users hourly varied significantly across months (H = 1707.27, N = 31517, p < 0.001, Fig 3B), with significantly fewer water users in the autumn and winter months of May–August than the summer months of December, January and February (post hoc Dunn’s test: p < 0.001).

### Daily patterns in shark sightings and recreational water use

#### Shark sightings

**Fig 4:**
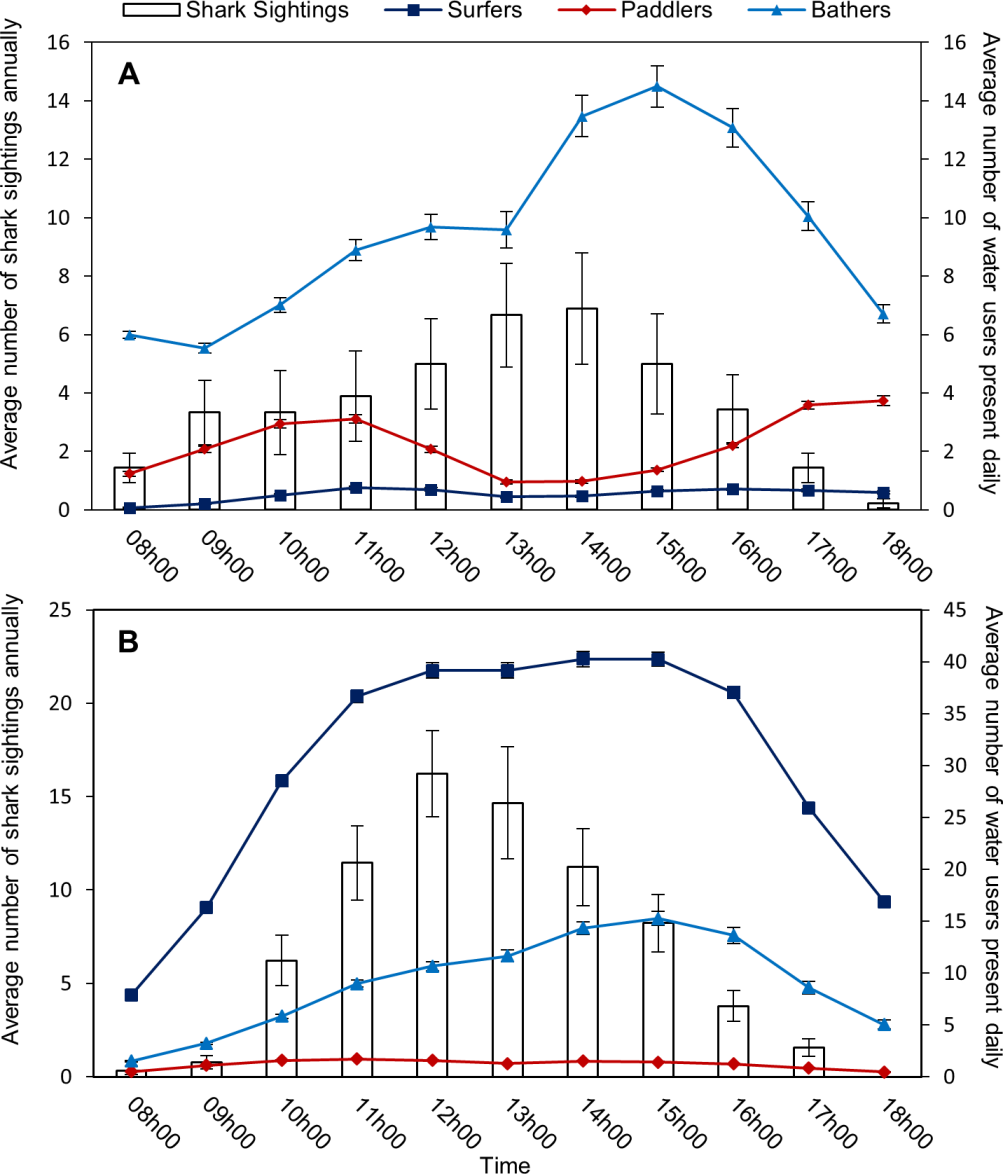
Average number (±SE) of shark sightings (bars) and the different water user groups (surfers–dark blue line, paddlers–red line, bathers–light blue line) across all study years at (A) Fish Hoek beach, and (B) Muizenberg beach.

The average number of shark sightings at Fish Hoek beach varied significantly with time of day (F = 26.37, n = 99, p = 0.003, Fig 4A), and was significantly lower for the hour of 18h00 relative to the midday hours of 13h00 and 14h00 (post hoc Tukey’s test: p < 0.05). The average number of shark sightings at Muizenberg beach also varied significantly with time of day (F = 74.81, n = 99, p < 0.001, Fig 4B), and was significantly higher in the hours between 11h00 and 14h00 (with a peak at 12h00) than in the early morning (08h00 and 09h00) and evening (18h00, post hoc Tukey’s test: p < 0.05).

#### Recreational water users

**Surfers:** The average number of surfers varied significantly throughout the day at Fish Hoek (H = 706.12, N = 29920, p < 0.001, Fig 4A), with two peaks in abundance between the hours of 10h00–12h00, and 15h00–17h00, during which there were significantly more surfers present than in the early morning (08h00 and 09h00) and evening (18h00, post hoc Dunn’s test: p < 0.05). The average number of surfers present at Muizenberg beach also varied significantly (H = 5912.69, N = 31517, p < 0.001, Fig 4B), and showed a significant peak (post hoc Dunn’s test: p < 0.001) in the hours between 11h00 and 16h00 than in the morning (08h00–10h00) and evening (17h00–18h00) (post hoc Dunn’s test: p < 0.05).

**Paddlers:** The average number of paddlers varied significantly throughout the day at Fish Hoek beach (H = 1475.91, N = 29920, p < 0.001, Fig 4A), with two peaks in abundance between the hours of 09h00–12h00, and 15h00–18h00, during which there were significantly more paddlers present than in the morning (08h00) and early afternoon (13h00–14h00, post hoc Dunn’s test: p < 0.001). At Muizenberg beach, the average number of paddlers also varied significantly (H = 1444.73, N = 31517, p < 0.001, Fig 4B), with fewer paddlers in the early morning (08h00) and evening (18h00) than the rest of the day (post hoc Dunn’s test: p < 0.001).

**Bathers:** The average number of bathers varied significantly throughout the day at Fish Hoek Beach (H = 811.66, N = 29920, p < 0.001, Fig 4A), with significantly more bathers present in the afternoon from 14h00 to 16h00 than the rest of the day (post hoc Dunn’s test: p < 0.05). The average number of bathers also varied significantly at Muizenberg (H = 2533.78, N = 31517, p < 0.001, Fig 4B), with a peak in abundance in the afternoon (14h00 and 15h00) relative to the rest of the day (post hoc Dunn’s test: p < 0.01).

### Impact of shark warnings on different water user categories

**Fig 5:**
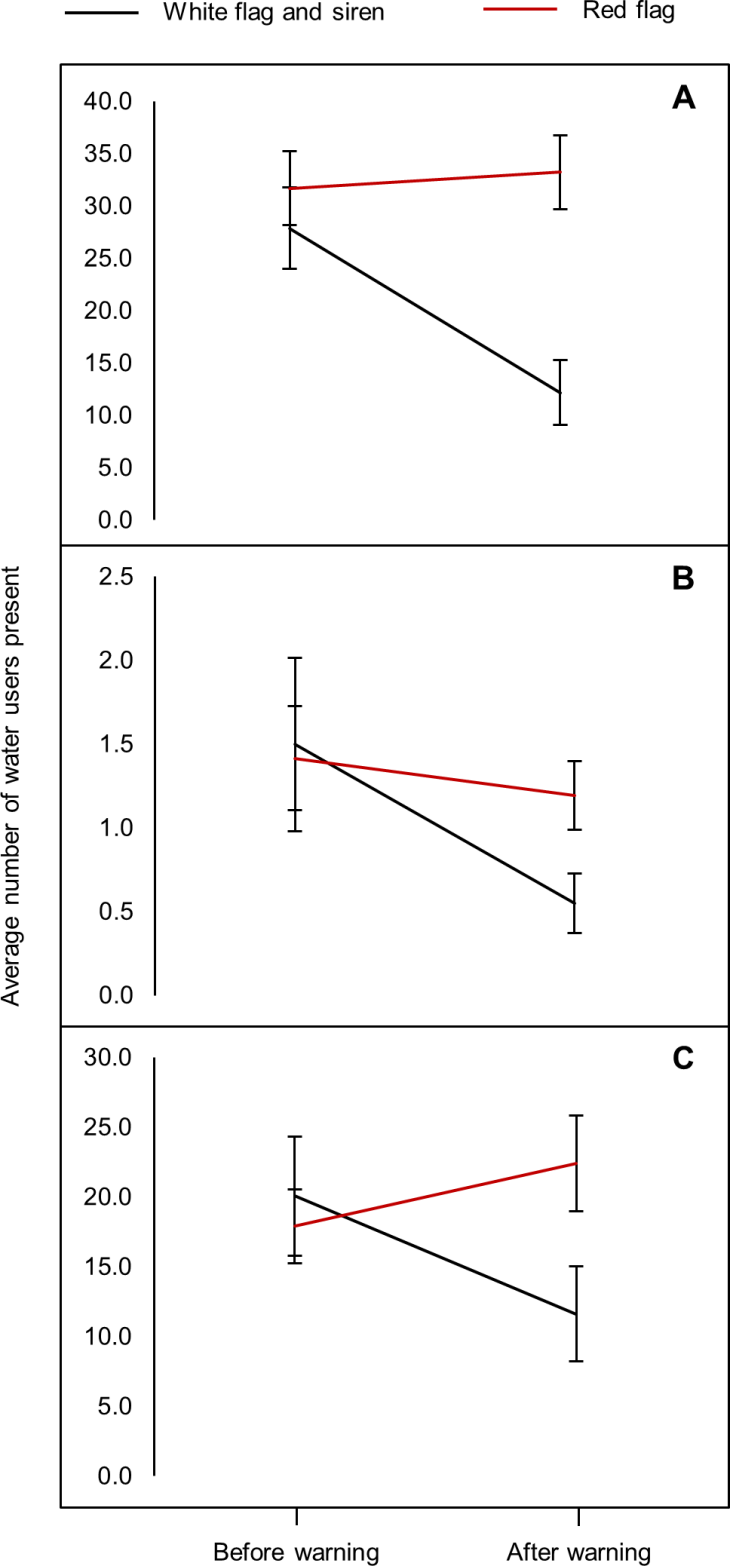
Average number (±SE) of water users, (A) surfers, (B) paddlers and (C) bathers, before and after the raising of a white flag with an accompanying siren (black line) vs. a red flag (red line) for both beaches across all study years.

Surfers, paddlers and bathers all exhibited a significant reduction in numbers following the raising of white flag accompanied by a siren to signal a beach closure (surfers: U = 1560.50, N_before_ = 71, N_after_ = 71, p < 0.0001, Fig 5A; paddlers: U = 1812,0, N_before_ = 71, N_after_ = 71, p < 0.001, Fig 5B; bathers: U = 1563.0, N_before_ = 310, N_after_ = 310, p < 0.0001, Fig 5C). In contrast, there was no significant change in the abundance of the different user groups following the raising of a red flag (surfers: U = 2805.5, N_before_ = 77, N_after_ = 77, p = 0.566, Fig 5A; paddlers: U = 2829.0, N_before_ = 77, N_after_ = 77, p = 0.58, Fig 5B; bathers: U = 2815,0, N_before_ = 77, N_after_ = 77, p = 0.59, Fig 5C).

### Impact of consecutive flag changes on water user abundance

**Fig 6:**
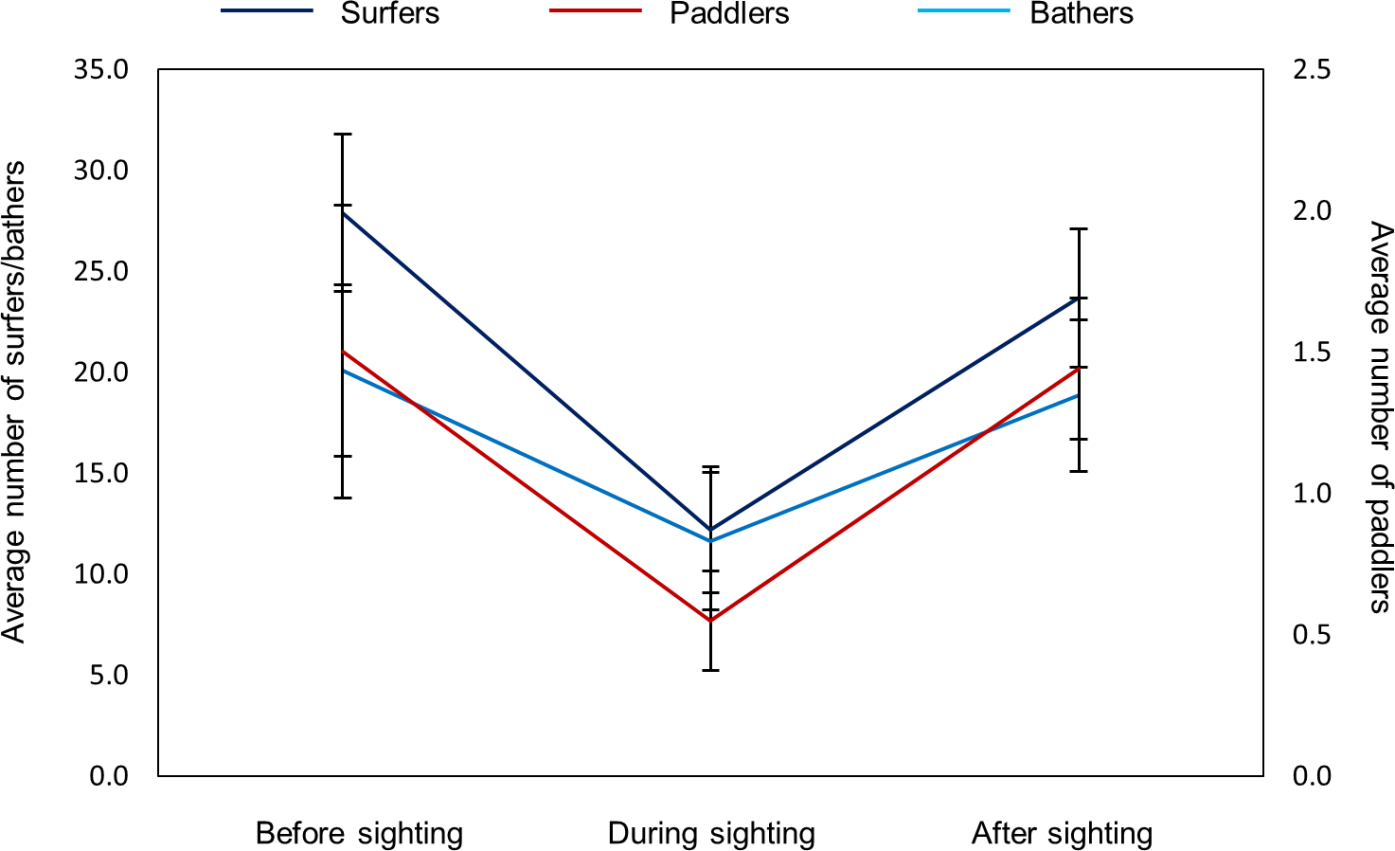
Average number (±SE) of water users from each category (surfers–dark blue line, paddlers–red line, bathers–light blue line) before, during and after a high risk shark sighting for both beaches across all study years.

There was a significant change in the number of water users present over three consecutive hours in which flag changes occurred from black/green to white to red (surfers: H = 21.26, N = 208, p < 0.001; paddlers: H = 13.51, N = 206, p = 0.001; bathers: H = 18.20, N = 208, p = 0.0001, Fig 6). The first change from black/green to white resulted in a significant reduction in the abundance of all water user categories (post hoc Dunn’s test, surfers: p < 0.001; paddlers: p = 0.021; bathers: p < 0.001). However, there was then a significant increase in all water user categories in response to the flag change from white to red the following hour (post hoc Dunn’s test, surfers: p = 0.0024; paddlers: p = 0.018; Bathers: p = 0.0037). The number of water users present while the red flag was raised did not differ significantly from that prior to the sighting when the flag colour was green/black (post hoc Dunn’s test, All groups: p = 0.999)

### Impact of a fatal attack on water user abundance

**Fig 7:**
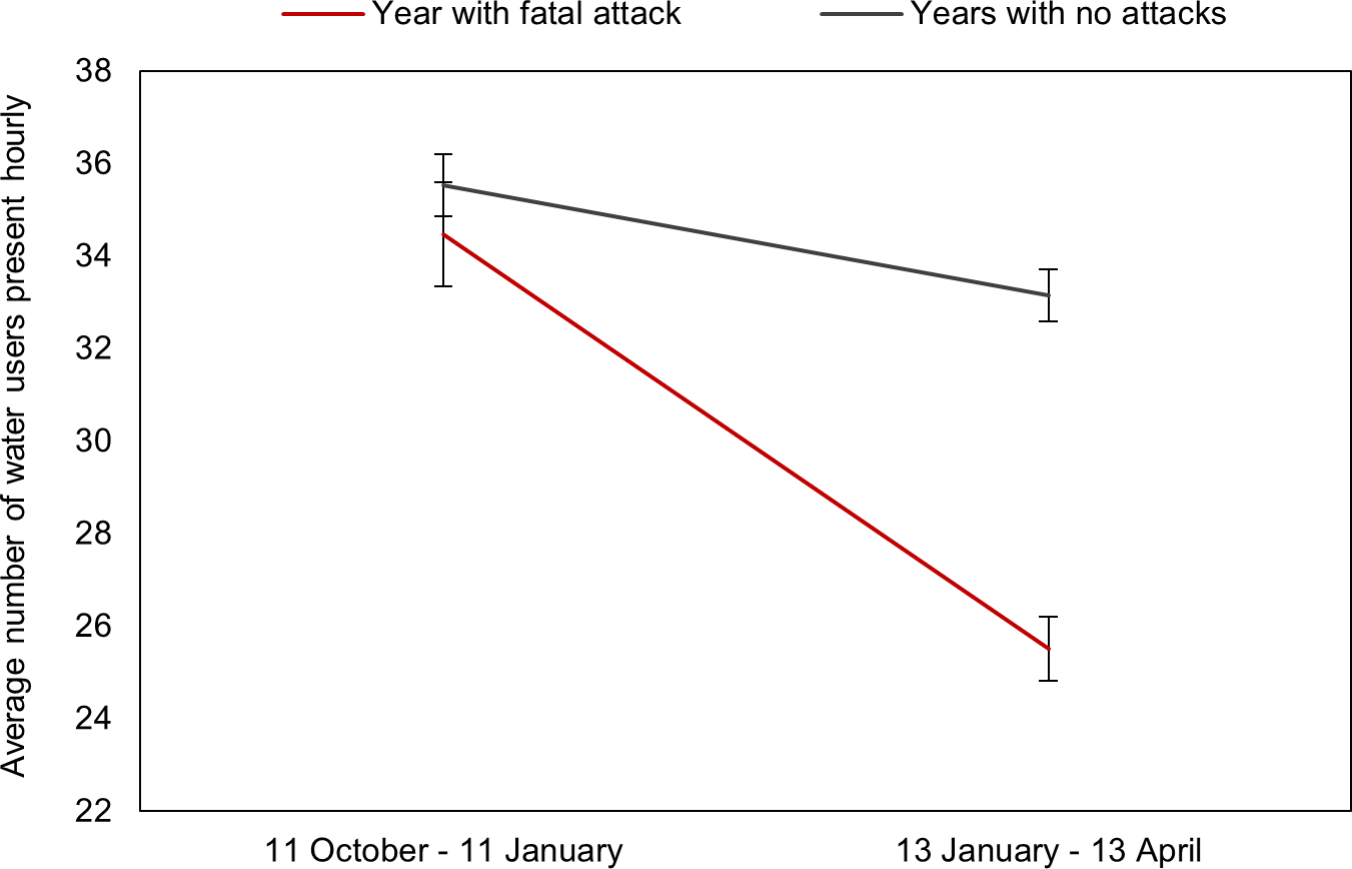
Change in the average number (±SE) of water users hourly at both beaches for the three months prior to a fatal shark attack versus the three months following the attack (red line), in comparison to the observed change in average hourly water user abundance over the same time period for years in which no attacks took place (grey line).

The average number of water users at both beaches was not significantly different over the period from 11 October 2009–11 January 2010 when compared to other years during which no attack occurred (U = 6164792, N_attack_ = 2040, N_no attack_ = 6218, p = 0.057, Fig 7). However, the average number of water users at both beaches was significantly lower in the three months following the fatal attack at Fish Hoek beach in 2010 compared to the same period in years during which no attack occurred (U = 7442908, N_attack_ = 1938, N_no attack_ = 8273, p < 0.0001).

## Discussion

### Temporal patterns in shark sightings and recreational water use

The number of shark sightings and water users peaked during the austral spring and summer months at Fish Hoek and Muizenberg, with few sightings in the winter months of May—August. The fact that the majority of the total sightings at both beaches were made in “poor visibility” conditions suggests that the observed decline in shark sightings during winter months was not a result of poor visibility, but an actual decrease in white shark presence in the inshore zone from May to August. This finding is supported by data from acoustic telemetry [29], which showed a marked absence of white sharks along the inshore areas of False Bay during the winter months, a time of year when white sharks typically aggregate at Seal Island in order to predate on young of the year Cape fur seals [29–31]. Peaks in fish abundance, combined with increasing difficulty in predating on older, more experienced juvenile seals at Seal Island are the likely drivers of increased white shark presence inshore during spring and summer [29–31]. The notable overlap between white sharks and water users in the inshore zone during the spring and summer months has important implications for human-wildlife conflict management. Analysis of 25 unprovoked shark attacks at Cape Town beaches from 1960–2005 showed that the highest number of attacks occurred in the month of September [32]. It is therefore imperative that the efficacy of the Shark Spotters program be maximised to prevent conflict during these times of year when overlap between water users and white sharks peaks in the inshore zone.

A common perception is that dawn and dusk are the riskiest times for water users because this is the time that many shark species are active [33]. Consequently, popular safety advice suggests avoiding these periods [34]. While this is true for white sharks attacking Cape fur seals (*Arctocephalus pusillus pusillus*) at Seal Island, False Bay [35], it does not seem to be the case for water users at Muizenberg or Fish Hoek. We found a marked diel trend in the extent of spatial overlap between white sharks and water users, with the number of shark sightings and water users peaking during the middle of the day to early afternoon at Fish Hoek and Muizenberg. These diel patterns in shark presence are supported by acoustic telemetry data, which indicate a peak in white shark presence at Muizenberg and Fish Hoek during the day (09h00–16h00) [29]. Indeed, an analysis of shark attack statistics at Cape Town beaches showed that all recorded attacks took place between 08h00–19h00, with the highest number occurring between 15h00–16h00 [32]. This highlights the need for understanding region- and species-specific shark behaviour for inclusion in local shark safety education and awareness campaigns. However, at the level of the individual, dawn and dusk remain high risk periods due to other factors, such as no shark spotters or life-guards on duty, low light which may increase the risk of misidentification of water users as a prey species by white sharks, and the low number of water users at these times which means individuals are more isolated and hence more at risk.

Despite this marked seasonal and diel overlap between water users and sharks at both beaches, shark attacks are extremely rare, averaging <1 per year over the last ten years. The rarity of shark incidents is a clear indication that white sharks are not actively targeting water users as a prey source. Rather, attacks are likely a consequence of the large number of recreational water users sharing the inshore zone with a large, opportunistic apex predator on a daily basis.

### Impact of shark warnings on different water user categories

The raising of a white flag accompanied by a siren resulted in a significant reduction in the number of water users across all categories, effectively reducing spatial overlap between people and sharks. In contrast, the raising of the red flag only (indicating “high shark alert”), in the absence of a siren, had no significant impact on any water user category. Initially it was thought that this may be solely due to water users being focused on incoming waves and hence not noticing the flag change on the beach in the absence of an auditory cue. However, the significant increase in water users that was observed after a flag change from white to red indicates that water users are also choosing to re-enter the water immediately after the white flag is removed, despite the red flag being in use. Although the red flag denotes a lower level of risk than the white flag and siren, it is still important that water users understand the increased risk of being in the surf zone when the red flag is raised.

It is important to understand the decision making of individual water users through a formal survey and, based on the outcomes of this, possible improvements to public education (especially through surf schools) on flag colour and risk can be made. However, the response to warnings will always be at the individual level. Thus, the acceptance of risk and ignoring of shark warnings by water users will remain an ongoing challenge to preventing incidents. For example, a shark attack that occurred at Fish Hoek beach in 2011 took place after the siren had been sounded while the white flag was in use. Despite the beach being closed with shark warnings in place, the victim chose to accept the risk and enter the water.

### Impact of a fatal attack on water user abundance

The occurrence of a fatal attack significantly reduced average water user abundance at both beaches for at least three months following the incident. A survey conducted following a non-fatal attack in 2011 concluded that public perceptions of sharks and their faith in the Shark Spotter program were not affected by the incident [36]. However, the significant decline in water user abundance for at least three months following the fatal attack in 2010 shows that these incidents have a marked effect on water user abundance and behaviour. This decline in water use has economic implications for local businesses that rely on tourism and recreational water use activities for income. Following the fatal attack in 2010, several tourism-based businesses in False Bay called for the local municipality to implement additional shark control measures, including the use of selected culling to reduce shark presence in the inshore zone. This illustrates that despite the rarity of shark bites, a single severe incident can have a notable influence on public risk perception, deterring recreational water users and impacting local businesses, which in turn can put tremendous pressure on local authorities to implement lethal control measures. It is therefore imperative that current shark safety and education strategies are optimised to prevent these incidents from occurring.

## Conclusions

There was marked overlap between water users and sharks in the inshore zone of False Bay, and consequently a high potential risk of conflict at popular recreational beaches such as Fish Hoek and Muizenberg. The immediate reduction of this overlap by means of a white flag, siren and subsequent beach closure indicated that Shark Spotters effectively reduced the risk of a shark incident for recreational water users. The lack of impact on water users by using the red flag needs to be further investigated in order to understand public risk perceptions and to recommend possible improvements to the program. This is important in order to prevent the occurrence and resultant negative repercussions of a shark incident on the local community and economy. Ultimately, the Shark Spotters program provides water users with information for balancing their recreational pursuits with their risk of encountering a shark and in doing so provides a sustainable non-lethal management option for ensuring coexistence of people and white sharks in the inshore region of False Bay.
